# Editorial: Novel biomarkers in acute aortic dissection

**DOI:** 10.3389/fcvm.2024.1457521

**Published:** 2024-07-17

**Authors:** Panagiota Georgiadou, Eftihia Sbarouni, John Elefteriades

**Affiliations:** ^1^ Division of Interventional Cardiology, Onassis Cardiac Surgery Center, Athens, Greece; ^2^ Aortic Institute at Yale – New Haven Hospital, Yale University School of Medicine, New Haven, CT, United States

**Keywords:** acute aortic dissection, biomarkers, diagnosis, prognosis, mortality

**Editorial on the Research Topic** Novel biomarkers in acute aortic dissection

Acute aortic dissection (AAD) is a lethal cardiovascular emergency with a mortality rate of 1%–2% per hour early after symptom onset (1, 2). Ninety-five per cent of AAD patients are asymptomatic before this occurs and even 20% of the symptomatic patients may have inconclusive signs and symptoms (1, 2). The biological basis and pathological processes of the aortic wall involved in AAD initiation and progression are not completely clear. Although a number of new biochemical markers have been assessed, little progress has been made in this field (3, 4).

The occurrence and the evolution of damage to aortic wall are mainly caused by subintimal inflammation, media degeneration, and apoptosis in response to internal and external factors. Systemic hypertension, bicuspid aortic valves (BAV), Marfan syndrome, and smoking are common risk factors for ascending aorta aneurysms and dissections (1, 2). Abnormal immune- inflammatory responses and altered metabolic effects have gained increasing attention recently. Genetic, inflammatory, metabolic, and immunological biomarkers are implicated in relevant pathways activated in the pathophysiology of AAD, contributing in different ways to the evolution of tissue damage (3, 4).

Over the past decades, great advancements have been achieved in biochemical sciences. Genomics, transcriptomics and proteomics, combined with the use of the appropriate bioinformatic analytic tools that directly reflect the physiological state of aortic disease, may help us to elucidate the mechanisms and accurately map the risk pathways to dissection (5, 6). Metabolomics and the differences in metabolic levels should be concomitantly used and integrated for screening, prompt diagnosis, disease monitoring and treatment (5, 6).

In this perspective, Wan et al. presented a rigorous bioinformatics analysis of several gene-expression databases to identify possible biomarkers associated with increased risk of thoracic aortic aneurysm and dissection (TAAD) with aging (Wan et al.). Aging-related differentially expressed genes (DEGs) were identified and in particular, the hypoxia inducible factor-1 (HIF-1) signaling pathway may play a significant role in TAAD and aging. The study also revealed that the aging-related genes MYC, IL6, HIF1A, ESR1 and PTGS2 could be useful biomarkers in aging-related TAAD (Wan et al.). The results point to some interesting pathways and gene expression changes, providing novel targets for TAAD diagnostics in the future. However, considering the complex role of HIF-1 in diverse cardiovascular diseases, the specificity of the results for aging-related TAAD needs further validation. Wet-lab research additionally would strengthen the findings of this study.

Hao et al. investigated potential metabolomic biomarkers for the identification and diagnosis of AAD in 20 hypertensive patients with AAD compared with 20 hypertensive patients without AAD (Hao et al.). There is a strong association between uncontrolled hypertension and AAD, which is mainly related to vascular biomechanics. Metabolites involved in lipid metabolism (fatty acid biosynthesis, biosynthesis of unsaturated fatty acids, and linoleic acid metabolism), carbohydrate metabolism (galactose, fructose, and mannose metabolisms) and membrane transport (ATP-binding cassette transporters) were found to differ significantly between groups (Hao et al.). Plasma hydrocortisone and dimethylglycine levels were significantly higher in AAD patients and demonstrated the highest AUC values (>0.92) in ROC curve analysis (Hao et al.). Despite the small sample size and the lack of a healthy control group, they provided new evidence for the exploration of biomarkers during the early clinical diagnosis of AAD.

Peng et al. studied the potential value of serum cell division cycle 42 and CD4+ T cell subsets for diagnosis and prognosis in 127 Stanford type B AAD patients (TBAD) compared to 30 healthy controls (Peng et al.). Serum CDC42 was reduced in TBAD patients vs. healthy controls and was independently correlated with lower risk of in-hospital mortality (*p* = 0.043) (Peng et al.). Serum CDC42 was negatively associated with Th17 cells (*p* = 0.001) (Peng et al.). Decreased CDC42 is associated with impaired vascular stiffness by suppressing actomyosin-contractility, leading to an increased risk of AAD and higher risk of mortality. Reduced CDC42 may enhance inflammatory cell infiltration, particularly up-regulating the expression of Th17 cells and further promoting aortic wall remodeling and the progression of AAD. Similarly, previous studies have shown elevated levels of Th17 cells in patients who died compared to survivors, suggesting these as substantial indicators of 30-day mortality risk in Stanford type A AAD patients (AUC = 0.741, CI: 0.624–0.867) (7). Taking into consideration the relatively small sample size, these findings may provide evidence in establishing a novel biomarker for the identification of dissection and cardiovascular mortality risk.

Staal et al. analyzed aortic specimens among three groups of patients with BAV (non-dilated, dilated and dissected aortas) using the novel technique of multiplex immunohistochemistry (Staal et al.). They found a 4-fold increase of lymphocytes and a 25-fold increase in B lymphocytes in the adventitia of dilated aortas compared to non-dilated aortas. Tertiary lymphoid structures with B cell follicles and helper T cell expansion were identified in dilated and dissected aortas (Staal et al.). Dilated aortas were associated with an increase in M1-like macrophages. Additionally, there was only mild media degeneration (27% in non-dilated specimens and 20% in dilated) with no significant differences in macrophage abundance regardless of the presence/absence of media degeneration. Although a control group of non-BAV cases is lacking, the crucial role of the complex interaction between the innate and the adaptive parts of the immune-inflammatory systems is indicated by the results of this study as well.

The findings of these four studies pave the way for novel avenues in aortic pathology research at the cellular and molecular levels. However, it is challenging to generalize their results due to the small sample sizes, and further validation with larger scale studies is needed. Distinguishing causal-risk vs. bystander-effect remains a major issue before diagnostic algorithms and medical therapies are introduced for clinical care.
